# THE ROLE OF NETWORKS IN TEACHING GERONTOLOGY THROUGH COMMUNITY ENGAGEMENT AND SERVICE-LEARNING PROJECTS

**DOI:** 10.1093/geroni/igz038.1983

**Published:** 2019-11-08

**Authors:** Tina M Kruger

**Affiliations:** Indiana State University, Terre Haute, Indiana, United States

## Abstract

As we seek to prepare the next generation of researchers, teachers, and service providers in the field of aging, it is vital that students garner experience interacting directly and appropriately with older adults. Community engagement and service-learning (CE/SL) are high impact pedagogical practices associated with enhanced learning, retention, and student success and can facilitate positive interactions across generations. Creating CE/SL opportunities requires authentic relationships with community members and organizations, allowing faculty, staff, and students the opportunity to network with a variety of individuals and agencies. In this presentation, we define CE/SL, examine the role of networks in creating CE/SL opportunities, and discuss challenges and threats to effective CE/SL projects. Tools and resources will be highlighted to provide guidance to those wishing to harness the power of networks to teach gerontology content while also working to have positive, lasting, and measurable impact on the communities in which they serve.

